# Imeglimin Improved Plasma Glucose Levels in Patients With Latent Autoimmune Diabetes of Adults: Report of 2 Cases

**DOI:** 10.1210/jcemcr/luad161

**Published:** 2023-12-19

**Authors:** Shuichi Okada, Kazuya Okada, Junichi Okada, Eijiro Yamada

**Affiliations:** Department of Diabetes, Soleiyu Asahi Clinic, Maebashi Gunma 371-0014, Japan; Department of Orthopedic Surgery, Tone Chuo Hospital, Numata Gunma 378-0012, Japan; Department of Medicine, Division of Endocrinology, Albert Einstein College of Medicine, Bronx, NY 10461, USA; Department of Medicine and Molecular Science, Gunma University Graduate School of Medicine, Maebashi Gunma 371-8511, Japan

**Keywords:** latent autoimmune diabetes of adults, imeglimin, glutamic acid decarboxylase antibody, diabetic ketoacidosis, glycated hemoglobin

## Abstract

Imeglimin has not been well studied as an oral agent for the treatment of latent autoimmune diabetes of adults (LADA). We treated 2 cases of LADA with imeglimin. The case 1 patient was originally diagnosed with type 2 diabetes (T2D) at age 50 years and was treated with sulfonylurea, biguanide, canagliflozin, imeglimin, and dulaglutide. Before imeglimin, his glycated hemoglobin A_1c_ (HbA_1c_) change was 94.0 mmol/mol (8.6%) in November 2022, but it dropped to 71.0 mmol/mol (6.5%) in May 2023 after imeglimin was added. The case 2 patient was originally diagnosed with T2D when she was aged 48 years. She was treated with vildagliptin, biguanide, luseogliflozin, and imeglimin. Her HbA_1c_ before imeglimin was 92.9 mmol/mol (8.5%) in January 2023, which decreased to 75.4 mmol/mol (6.9%) in July 2023 after imeglimin was added. Although imeglimin has not been approved for treating type 1 diabetes and LADA, adding imeglimin to the current medication was effective in improving and controlling the patients’ plasma glucose.

## Introduction

Imeglimin is a novel oral agent for the treatment of type 2 diabetes (T2D). Imeglimin's mechanism of action has dual effects: (a) increased glucose-stimulated insulin secretion and preservation of β-cell mass; and (b) enhanced insulin action, including the potential for inhibiting hepatic glucose output and improving insulin signaling both in the liver and skeletal muscle [1].

Latent autoimmune diabetes of adults (LADA) is a form of diabetes mellitus (DM) with features of both type 1 diabetes (T1D) and T2D. In Japan, LADA is referred to as slowly progressive insulin-dependent type 1 DM (SPIDDM). The American Diabetes Association classifies LADA as T1D that evolves more slowly than the classic disease and does not recognize it as a specific type of DM, while the World Health Organization termed LADA as “slowly evolving immune-related diabetes.”

LADA is a disease of adults and the Immunology for Diabetes Society (IDS) has specified 3 criteria for its diagnosis:

1. Age greater than 35 years
2. Positive autoantibodies to islet β cells
3. Insulin independence for at least the initial 6 months after initial diagnosis.

The use of oral agents such as imeglimin for treating LADA has not been thoroughly studied [2]. We present 2 cases of LADA treated with imeglimin.

This study was performed in line with the principles of the Declaration of Helsinki. Approval was granted by the review board of Soleiyu Asahi Clinic (November 5, 2022/No. 2023-C-1).

## Case Presentation

### Case 1

In June 2023, an 81-year-old male patient (body height: 163.0 cm, body weight: 56.7 kg, body mass index [BMI]: 21.3) was transferred to our clinic to continue his DM treatment. His previous history was unremarkable. His mother was being treated for T2D and he was also originally diagnosed with T2D when he was aged 50 years.

On the first visit to our clinic, the patient's casual peripheral blood laboratory findings were as follows: aspartate transaminase (AST) 18 IU/L (normal range, 10-40 IU/L), alanine transaminase (ALT) 13 IU/L (normal range, 5-45 IU/L), γ-glutamyl transpeptidase (GTP) 11 IU/L (normal range, 0-79 IU/L), triglycerides (TGs) 3.18 mmoL/L (282 mg/dL) (normal range < 1.98 mmoL/L [<175 mg/dL]), high-density lipoprotein (HDL) 0.98 mmoL/moL (38 mg/dL) (normal range, 1.0-2.1 mmoL/moL [40-80 mg/dL]), low-density lipoprotein (LDL) 1.73 mmoL/L (67 mg/dL) (normal range, 1.81-3.59 mmoL/L [70-139 mg/dL]), blood urea nitrogen (BUN) 5.5 mmoL/L (15.4 mg/dL) (normal range, 2.86-7.14 mmoL/L [8.0-20.0 mg/dL]), serum creatinine (Cre) 97.2 μmol/L (1.1 mg/dL) (normal range, 57.5-96.4μmol/L [0.65-1.09 mg/dL]), estimated glomerular filtration rate (eGFR) 50.0 mL/min/1.73m^2^, uric acid (UA) 327.1 μmol/L (5.5 mg/dL) (normal range, 214.1-416.4 μmol/L [3.6-7.0 mg/dL]), sodium (Na) 142 mmoL/L (142 mEq/L) (normal range, 135-145 mmoL/L [135-145 mEq/L]), potassium (K) 5.0 mmoL/L (5.0 mEq/L) (normal range, 3.5-5.0 mmoL/L [3.5-5.0 mEq/L]), chloride (CL) 106 mmoL/L (106 mEq/L) (normal range, 98-108 mmoL/L[98-108 mEq/L]), serum amylase 72 IU/L (normal range, 39-134 IU/L), white blood cell count (WBC) 6790/mL (normal range, 3500-9700/mL), red blood cell count (RBC) 552 × 104/mL (normal range, 438-577/mL), Hb 138 g/L (13.8 g/dL) (normal range, 136-183 g/L [13.6-18.3 g/dL]), hematocrit (Hct) 0.439/L (43.9%) (normal range, 0.405-0.519/L [40.5%-51.9%]), platelet 20.7 × 104/mL (normal range, 14.0-37.9/mL), urinary albumin/creatinine ratio (UACR) 28.9 mg/g (normal range, < 30 mg/g), casual plasma glucose (PG) 5.88 mmoL/L (106 mg/dL), and casual serum insulin 8.2 μU/mL. His urine was negative for glucose, protein, and ketone body, and RBCs and WBCs were not detected in the urine sediment.

Chest x-ray examination and electrocardiogram were normal. He had simple diabetic retinopathy and bilateral light numbness in the lower limbs due to diabetic neuropathy.

### Case 2

In June 2023, a 55-year-old female patient (body height: 158.0 cm, body weight: 61.2 kg, BMI: 24.5) was also transferred to our clinic to continue her DM treatment. She had previously been treated for hypertension and hypercholesteremia using bisoprolol fumarate and rosuvastatin calcium, respectively. In her family, her daughter is being treated for T1D. She was diagnosed with T2D at age 48 years.

On her first visit to our clinic, her casual peripheral blood laboratory findings were as follows: AST: 14 IU/L, ALT: 17 IU/L, γ-GTP: 13 IU/L, TGs: 0.94 mmoL/L (83 mg/dL), HDL: 1.6 mmoL/L (62 mg/dL), LDL: 2.48 mmoL/L (96 mg/dL), BUN: 5.14 mmoL/L (14.4 mg/dL), Cre: 45.1 μmoL/L0.51 mg/mL, eGFR: 114.0 mL/min/1.73m^2^, UA: 160.5 μmoL/L (2.8 mg/dL), Na: 142 mmoL/L (142 mEq/L), K: 3.9 mmoL/L (3.9 mEq/L), CL: 103 mmoL/L (103 mEq/L), serum amylase: 60 IU/L, WBC: 5160/mL, RBC: 459 × 104/mL, Hb: 133 g/L (13.3 g/dL), Hct: 0.405/L (40.5%), platelet: 30.1 × 104/mL, UACR: 13.9 mg/g; casual PG: 148 mg/dL, and casual serum insulin: 12.0 μU/mL. The urinalysis result was negative for glucose, protein, and ketone body, and no RBCs or WBCs were detected in the urine sediment.

A chest x-ray examination and electrocardiogram revealed no abnormalities. She did not have diabetic neuropathy and retinopathy.

## Diagnostic Assessment

### Case 1

The patient had never had diabetic ketoacidosis and had never been treated with insulin until today. A screening examination in our clinic revealed that his antiglutamic acid decarboxylase (GAD) antibody level was 14.3 U/mL (normal range <5.0 U/mL). According to the diagnosis criteria for LADA as described earlier, we rediagnosed his diabetes as LADA.

### Case 2

The patient had no diabetic ketoacidosis and was never administered insulin. Screening examination in our clinic revealed that her anti-GAD antibody level was 111.5 U/mL (<5.0 U/mL). According to the diagnosis criteria for LADA as described earlier, we rediagnosed his diabetes as LADA.

## Treatment

### Case 1

The patient was treated with sulfonylurea (5.0 mg/day), biguanide (1000 mg/day), canagliflozin (100 mg/day), imeglimin (2000mg/day), and dulaglutide. He received suggestions and advice regarding necessary diet and lifestyle modifications by a registered nurse, a registered dietician, and his physician when he visited our clinic.

### Case 2

The patient was treated with vildagliptin (100 mg/day), biguanide (1000 mg/day), luseogliflozin (5 mg/day), and imeglimin (2000 mg/day). She received suggestions and advice regarding necessary diet and lifestyle modifications by a registered nurse, a registered dietician, and her physician when he visited our clinic.

## Outcome and Follow-up

### Case 1

**Figure 1.**
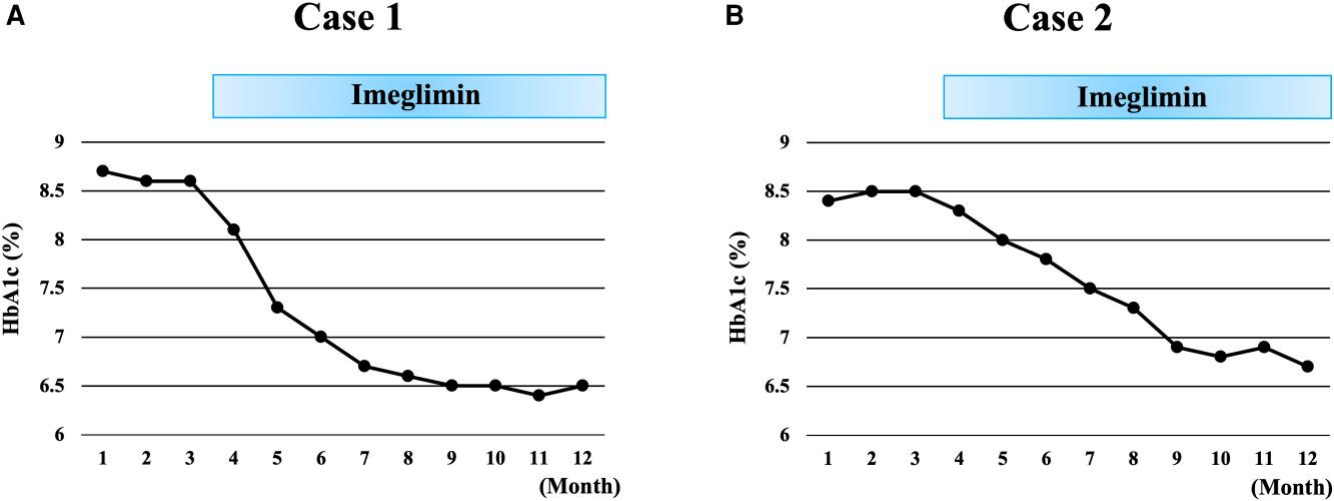
A, Changes in HbA_1c_ in case 1. In the line graph, the Y axis represents the HbA_1c_ (%) and the X axis represents the month of hospital visits. The patient had been administered sulfonylurea (5.0 mg/day), biguanide (1000 mg/day), canagliflozin (100 mg/day), and dulaglutide. Imeglimin was used, as indicated by the blue box. HbA_1c_, glycated hemoglobin A_1c_. B, Changes in HbA_1c_ in case 2. In the line graph, the Y axis represents the HbA_1c_ (%), and the X axis represents the month of hospital visits. The patient had been administered vildagliptin (100 mg/day), biguanide (1000 mg/day), and luseogliflozin (5 mg/day). Imeglimin was used, as indicated by the blue box. HbA_1c_, glycated hemoglobin A_1c_.

The result of the HbA_1c_ test is depicted in Fig. 1A. Before imeglimin was added, the patient’s HbA_1c_ level was 82.0 mmoL/moL (7.5%) in May 2022, 85.3 mmoL/moL (7.8%) in September 2022, and 94.0 mmoL/moL (8.6%) in November 2022. Imeglimin (2000mg/day) was added to the current medication in November 2022. Thereafter, the HbA_1c_ level dropped from 88.5 mmoL moL (8.1%) in January 2023 to 79.8 mmoL/moL (7.3%) in February 2023, 76.5 mmoL/moL (7.0%) in March 2023, 72.1 mmoL/moL (6.6%) in April 2023, 71.0 mmoL/moL (6.5%) in May 223, 71.0 mmoL/moL (6.5%) in June 2023, and 70.0 mmoL/moL (6.4%) in July the same year. Thus, imeglimin effectively improved and stabilized his plasma glucose.

### Case 2

The result of the patient’s HbA_1c_ test is depicted in Fig. 1B. Before imeglimin was added to her current medication, her HbA_1c_ level was 91.8 mmoL/mol (8.4%) and 92.9 mmoL/moL (8.5%) in November and December 2022, respectively, and 92.9 mmoL/moL (8.5%) in January 2023. In January 2023, imeglimin (2000mg/day) was added to her medication. Thereafter, her HbA_1c_ level dropped to 90.7 mmol/moL (8.3%) in February 2023, 87.4 mol/moL (8.0%) in March 2023, 84.3 mmoL/moL (7.7%) in April 2023, 82.0 mmoL/moL (7.5%) in May 2023, 79.8 mmoL/moL (7.3%) in June 2023, and 75.4 mmoL/moL (6.9%) in July 2023. Thus, imeglimin also effectively improved plasma glucose control in this patient.

## Discussion

LADA is autoimmune diabetes that begins in adulthood and does not require insulin for glycemic control for at least the first 6 months after diagnosis [3]. Although LADA shares genetic, immunologic, and metabolic features with both T1D and T2D, it has recently been reported to be less immunogenic than T1D [4].

Therapy that preserves pancreatic β-cell function is a top priority in the treatment of LADA, and insulin has been the treatment of choice. Studies have shown preserved pancreatic β-cell function, as evidenced by a sustained stimulated C-peptide response, normal HbA_1c_ levels, and a decrease in autoantibody concentrations [5]. On the other hand, sulfonylureas are a poor choice for LADA because they deplete β cells of insulin, as evidenced by falling C-peptide levels, persistence of antibodies, and earlier progression to requiring insulin treatment. Although metformin may initially improve glycemic control in LADA patients with higher BMI, it cannot solely achieve the second and more important goal of preserving pancreatic β-cell function or delaying its destruction. Thiazolidinediones have anti-inflammatory effects on pancreatic β cells that can prolong their survival and be beneficial if used in the earlier stage of LADA. Dipeptidyl-peptidase 4 inhibitors have shown promise in preserving pancreatic β-cell function in LADA, either alone or in combination with insulin. A study using the glucagon-like peptide 1 receptor agonist dulaglutide found that LADA patients had lower HbA_1c_ levels and improved pancreatic β-cell function, with results comparable to T2D [6]. Although the use of sodium and glucose cotransporter 2 inhibitors in LADA patients has not been well studied, they are not recommended due to reports of euglycemic ketoacidosis [7]. Imeglimin has not been approved for the treatment of T1D and LADA and has not been well studied. However, based on its mechanism of action, imeglimin could be expected to preserve pancreatic β-cell function in LADA patients [1].

Although both patients were previously treated as having T2D, we rediagnosed them as LADA based on the IDS diagnosis criteria [3]. However, there is a possibility that both cases could be T2D with positive GAD antibody [8].

Besides the addition of imeglimin, their medication was not changed but their HbA_1c_ level improved month to month after imeglimin was added. Hence, imeglimin significantly improved plasma glucose control in LADA patients. So far, the addition of imeglimin has had no adverse effect on either patient.

## Learning Points

- LADA is a form of diabetes mellitus with features of both T1D and T2D.
- Therapy that preserves pancreatic β-cell function is a top priority in the treatment of LADA.
- Imeglimin is a novel oral agent for the treatment of T2D.
- The use of imeglimin for treating LADA has not been thoroughly studied.
- Imeglimin significantly improved plasma glucose control in LADA patients.

## Contributors

S.O. is responsible for the patients’ clinical care. S.O., K.O., J.O., and E.Y. contributed to the analysis of data and writing of the case report. All authors read and approved the final manuscript.

## Data Availability

The data sets generated or analyzed during the current study are available from the corresponding author on reasonable request.

## Abbreviations

ALT: alanine transaminase
AST: aspartate transaminase
BMI: body mass index
BUN: blood urea nitrogen
CL: chloride
Cre: serum creatinine
DM: diabetes mellitus
eGFR: estimated glomerular filtration rate
GAD: glutamic acid decarboxylase
GTP: γ-glutamyl transpeptidase
Hb: hemoglobin
HbA_1c_: glycated hemoglobin A_1c_
Hct: hematocrit
HDL: high-density lipoprotein
IDS: Immunology for Diabetes Society
K: potassium
LADA: latent autoimmune diabetes of adults
LDL: low-density lipoprotein
Na: sodium
PG: plasma glucose
RBC: red blood cell count
T1D: type 1 diabetes
T2D: type 2 diabetes
TGs: triglycerides
UACR: urinary albumin/creatinine ratio
WBC: white blood cell count
